# The Disinfection of Infected Wounds by Means of Septic Materials

**Published:** 1896-12

**Authors:** 


The Disinfection of Infected Wounds by Means of Septic Materials. The question whether an infected wound can be perfectly cleansed by means of disinfection is, viewed from a practical point of view, according to the statement of Schimmelbusch, of relatively little importance. Operators who do not use these means of cleansing obtain the same results in their operations and dressings as those who apply antiseptics. To what extent the cleansing of an infected wound with a disinfecting fluid is of avail is clearly illustrated by an experiment. Treat a number of wounds, which have been infected with the same microbe, with and without antiseptics, and observe closely the results of both methods. In the first place, a wound in a white mouse which is infected with anthrax-bacillus causes the death of the animal even when the wound has been cleansed immediately with strong antiseptics. It is also impossible to prevent by these therapeutics the death of white rabbits which have been infected with streptococci. This is due to the fact that the microbes penetrate into the tissues very rapidly and so are not affected by the disinfectant. Schimmelbusch found that white mice which had been infected on the end of the tail with splenic-fever bacilli always died in ten minutes unless the tail was amputated within ten minutes after infection.—*Veearts. N. Bladen*, Deel ix., aflevering 4.

